# Natural History of the Bruise: Formation, Elimination, and Biological Effects of Oxidized Hemoglobin

**DOI:** 10.1155/2013/703571

**Published:** 2013-05-16

**Authors:** Viktória Jeney, John W. Eaton, György Balla, József Balla

**Affiliations:** ^1^Department of Medicine, University of Debrecen, Debrecen 4012, Hungary; ^2^MTA-DE Vascular Biology, Thrombosis and Hemostasis Research Group, Hungarian Academy of Sciences, Debrecen 4012, Hungary; ^3^Department of Medicine, James Graham Brown Cancer Center, University of Louisville, Louisville, KY 40059, USA; ^4^Department of Pediatrics, University of Debrecen, Debrecen 4012, Hungary

## Abstract

Numerous disease states are associated with hemolysis or hemorrhage. Because red cells in the extravascular space tend to lyse quickly, hemoglobin (Hb) is released and is prone to autoxidation producing MetHb. Inorganic and organic peroxides may convert Hb and MetHb to higher oxidation states such as ferrylHb. FerrylHb is not a single chemical entity but is a mixture of globin- and porphyrin-centered radicals and covalently cross-linked Hb multimers. Oxidized Hb species are potent prooxidants caused mainly by heme release from oxidized Hb. Moreover, ferrylHb is a strong proinflammatory agonist that targets vascular endothelial cells. This proinflammatory effect of ferrylHb requires actin polymerization, is characterized by the upregulation of proinflammatory adhesion molecules, and is independent of heme release. Deleterious effects of native Hb are controlled by haptoglobin (Hp) that binds cell-free Hb avidly and facilitates its removal from circulation through the CD163 macrophage scavenger receptor-mediated endocytosis. Under circumstances of Hb oxidation, Hp can prevent heme release from MetHb, but unfortunately the Hp-mediated removal of Hb is severely compromised when Hb is structurally altered such as in ferrylHb allowing deleterious downstream reactions to occur even in the presence of Hp.

## 1. Introduction

The red cell is usually a blessing but sometimes a curse. The normal red cell efficiently binds oxygen from the atmosphere, delivers it to the tissues, and helps remove the product of metabolic combustion, carbon dioxide. Nice. But the same cells can be involved in pathophysiologic mischief upon hemorrhage or intravascular hemolysis. Once outside the vascular system, red cells quickly burst releasing free hemoglobin (Hb). That Hb is prone to spontaneous oxidation (as the scientists trying to develop Hb-based blood substitutes have repeatedly discovered). Even worse, the Hb may be converted to higher oxidation states such as ferrylHb which have potent proinflammatory and prooxidant effects, either directly or via the release of heme which itself is highly prooxidant. Here, we briefly describe what is known regarding the natural mechanisms which have evolved to control this cell-free hemoglobin and what is being learned about the biologic effects of Hb once it is released from the red cell in pathologic states.

## 2. Antioxidant Network of Erythrocytes

Hemoglobin (Hb) autoxidation is the main source of reactive oxygen species inside erythrocytes. Taking into consideration the high intraerythrocytic concentration of Hb—around 5 mM as tetramer—even a slow rate of autoxidation could generate substantial amounts of ROS. To cope with this challenge, erythrocytes are equipped with highly effective antioxidant defenses [1]. This system includes enzymes such as Cu/Zn superoxide dismutase (SOD1) that convert superoxide anion to hydrogen peroxide (H_2_O_2_), catalase (Cat), glutathione peroxidase (Gpx-1), and peroxiredoxins (Prdx1 and Prdx2) which decompose H_2_O_2_ to H_2_O. Nonenzymatic scavengers such as glutathione also contribute to this protection.

Gene knock-out mouse models have been used to estimate the relative importance of these antioxidant defenses. Surprisingly, under steady-state conditions, mice deficient in either Gpx-1 [2] or Cat [3] have a normal phenotype. However, Cat seems to be essential for protection against elevated levels of H_2_O_2_ since Cat deficient erythrocytes are very sensitive to exogenous H_2_O_2_ [4]. It has been suggested that erythrocyte Cat might function as a “sink” for extra-erythrocytic H_2_O_2_ and thus protect somatic cells against exogenous oxidant challenge [5]. In contrast to *Gpx1* ^−/−^ or *Cat* ^−/−^ mice, *Prdx1* ^−/−^, *Prdx2* ^−/−^, and *SOD1* ^−/−^ mice exhibit impaired erythrocyte antioxidant defense. As a result, concentrations of ROS are elevated and accompanied by hemolytic anemia in *SOD1* ^−/−^ [6], *Prdx1* ^−/−^ [7], and *Prdx2* ^−/−^ mice [8].

The idea that Hb itself may have antioxidant properties and thus contribute to the antioxidant network of erythrocytes emerged recently. For example, the Hb *β*93 cysteine (*β*93Cys)—highly conserved in vertebrates—has been shown to scavenge superoxide anion produced in the heme pocket of the *β*-chain of Hb [9]. This reaction may be beneficial as it decreases the rate of Hb autoxidation and reduces heme degradation attributed to the reaction of superoxide with the heme [9]. The contribution of this Hb residue to erythrocyte antioxidant protection might be considerable because of its high concentration. Knock-in mouse models—in which erythrocytes contained wild-type human Hb or human Hb in which the *β*93 cysteine residue was replaced with Ala—were generated to directly explore the role of the conserved *β*93Cys [10]. While the authors did not observe hemolysis or hemolytic disorders in the *β*93Ala mice, they found that *β*93Ala Hb has higher reactivity toward H_2_O_2_ than *β*93Cys Hb [11]. When *β*93Ala Hb was reacted with H_2_O_2_, elevated formation of high molecular weight, presumably cross-linked Hb species were observed, suggesting that the *β*93Cys residue plays a protective role in the metabolism of reactive species produced by erythrocytes under stress conditions [11]. This notion was further supported by experiments in which oxidative stress was engendered *in vivo* in mice by challenging them with lipopolysaccharide (LPS). The authors found that LPS provoked more pronounced lung injury and a greater degree of hypotension in *β*93Ala versus *β*93Cys mice. These effects were accompanied by elevated level of erythrocyte reactive oxygen species [11].

Finally, the erythrocyte enzyme, NADH methemoglobin (MetHb) reductase (Aka diaphorase I) also contributes to the antioxidant network of the erythrocyte. Continuous exposure of erythrocyte Hb to oxygen leads to the oxidation of ferrous (Fe^2+^) heme to ferric (Fe^3+^) heme. The resulting MetHb is unable to carry oxygen. The function of MetHb reductase is to restore oxygen-binding ability of Hb by reducing ferric ion into ferrous ion (reviewed in [12]).

## 3. Oxidation of Hb

Under normal conditions, a very small amount of the oxygen attached to the Hb heme iron dissociates as superoxide anion leaving ferric heme. This explains why, in normal people, 1-2% of Hb is present as MetHb (1). Dismutation of superoxide anion by SODs generates H_2_O_2_. Numerous *in vitro* studies have been performed to describe the peroxide-mediated oxidation of Hb using purified human Hb.

Peroxides like H_2_O_2_ may trigger a two-electron oxidation of Hb producing ferryl (Fe^4+^=O^2-^) Hb (2), whereas the reaction of MetHb with H_2_O_2_ yields ferrylHb radical (Hb^∙+^(Fe^4+^ = O^2−^)) in which the unpaired electron is associated with the globin or the porphyrin ring (3) [13–16]

(1) $$\begin{array}{c} \text{Hb}\left({\text{Fe}}^{2+}\right){\text{O}}_{2}\to \text{Hb}\left({\text{Fe}}^{3+}\right)+{{\text{O}}_{2}}^{∙-} \end{array}$$

(2) $$\begin{array}{c} \text{Hb}\left({\text{Fe}}^{2+}\right){\text{O}}_{2}+{\text{H}}_{2}{\text{O}}_{2}\to \text{Hb}\left({\text{Fe}}^{4+}={\text{O}}^{2-}\right)+{\text{H}}_{2}\text{O}+{\text{O}}_{2} \end{array}$$

(3) $$\begin{array}{c} \text{Hb}\left({\text{Fe}}^{3+}\right)+{\text{H}}_{2}{\text{O}}_{2}\to {\text{Hb}}^{∙+}\left({\text{Fe}}^{4+}={\text{O}}^{2-}\right)+{\text{H}}_{2}\text{O} \end{array}$$

The high-valence iron compounds are reactive intermediates and decay by several routes [17]. FerrylHb can initiate further production of globin radicals via an intramolecular electron transfer between the ferryl iron and specific amino acid residues of the globin chains resulting in the formation of MetHb globin radical (3). Radical/radical termination of globin- and porphyrin-centered radicals leads to the production of globin-globin (4) or porphyrin-globin cross-links

(4) $$\begin{array}{c} \text{Hb}\left({\text{Fe}}^{4+}={\text{O}}^{2-}\right)+2{\text{H}}^{+}\to {\text{Hb}}^{∙+}\left({\text{Fe}}^{3+}\right)+{\text{H}}_{2}\text{O} \end{array}$$

(5) Hb∙+(Fe3+)+Hb∙+(Fe3+)→(Fe3+)Hb+–Hb+(Fe3+)

Considerable effort has been made to explore the mechanism via which the globin radicals are formed and where they are located within the protein. Several amino acids on both *α*- and *β*-globin chains were identified as targets of H_2_O_2_-triggered oxidation. When Hb was reacted with H_2_O_2_ in the presence of the spin trap 5,5-dimethyl-1-pyrroline N-oxide (DMPO), several DMPO adducts were formed [18, 19]. Identification of the precise amino acid residues trapped by the DMPO by mass spectrometry revealed that radical formation occurs on *β*Cys93, *α*Tyr24, *α*Tyr42, and *α*His-20 residues of the globin chains [18]. As part of this latter work, the authors showed that in the absence of a spin trap, the globin-centered radicals can decay by formation of cross-links between the globin chains [18]. Later investigations identified key amino acids that appear to be highly susceptible to H_2_O_2_-mediated oxidation [15]. During H_2_O_2_-triggered Hb oxidation, *β*Cys93 and *β*Cys112 residues are irreversibly oxidized to cysteic acids, while oxidative modification of *β*Trp-15 and *β*Met-55 also occurs [15]. These processes might contribute to the loss of *α*-helical structure of the *β* chain surrounding the heme pocket and eventually lead to the deformation and collapse of the *β* chains [15].

A number of studies have examined whether H_2_O_2_-mediated Hb oxidation occurs in intact erythrocytes despite the above-mentioned highly active antioxidant defense systems. Giulivi and Davies provided the first evidence for the formation of ferrylHb in intact red blood cells [20]. Later, the same group detected dityrosine in intact red blood cells exposed to H_2_O_2_ and provided a mechanism for its formation. They concluded that dityrosine is a hallmark of globin-centered tyrosyl radical formation and subsequent intermolecular cross-linking [21, 22].

Besides H_2_O_2_, oxidized lipids are very important triggers of Hb oxidation. For example, we showed that lipid hydroperoxides of atheroma lipids convert Hb to MetHb [23]. Furthermore, interactions between lipid hydroperoxides of oxLDL or atheroma lipids result in the formation of ferrylHb and covalently cross-linked Hb species [23].

### 3.1. Hb Oxidation *In Vivo*

Whether oxidation of Hb leading to the formation of high-valence heme/iron compounds and globin radicals occurs *in vivo* was a subject of debate until recently. These products are transiently formed and can initiate a range of oxidative reactions with similar reactivity to the hydroxyl radical. The first piece of evidence that the above-mentioned reactions occur *in vivo* came from the study of Svistunenko et al., in which the authors could detect ferrylHb globin-centered radicals in normal human blood by electron paramagnetic resonance spectroscopy [24]. Furthermore, covalently modified heme in Hb was detected by HPLC in normal human blood [25]. In the latter study, the authors showed that the concentration of covalently modified heme in Hb is increased by exercise, a surrogate model of acute oxidative stress [25].

Oxidation of Hb has been shown in certain pathological conditions. For example, heme-protein cross-linked Hb was detected in cerebrospinal fluid following subarachnoid hemorrhage by reverse phase HPLC [26]. Moreover, recently we found that the content of dityrosine is elevated and globin-globin cross-linked Hb multimers are present in complicated atherosclerotic lesions in humans [23].

## 4. Control of Free Hb and Hb-Derived Heme

**Figure 1:**
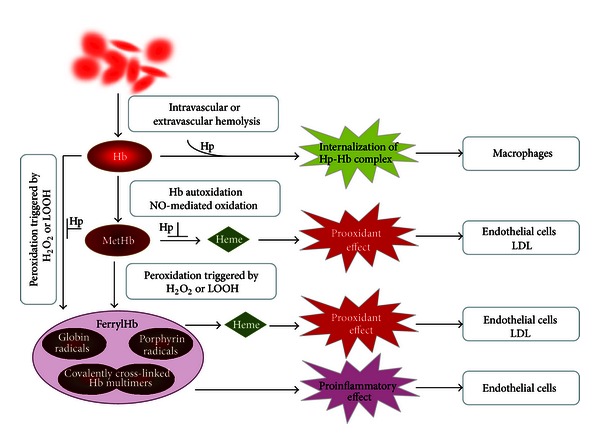
Schematic representation of hemoglobin oxidation and the different biological effects of oxidized Hb species. Hb is released from red blood cells following intra- or extravascular hemolysis. Hb can undergo spontaneous oxidation, or, alternatively, nitric oxide (NO) can trigger Hb oxidation to MetHb. Peroxidation of Hb and MetHb by H_2_O_2_ or lipid hydroperoxides (LOOH) leads to the formation of ferrylHb, that is, a mixture of globin radicals, porphyrin radicals, and covalently cross-linked Hb multimers. Haptoglobin (Hp) binds extracellular Hb and facilitates its internalization by macrophages. MetHb and ferrylHb can release heme and induce oxidative modification of lipids such as low-density lipoprotein (LDL), sensitizing cells to oxidant-mediated killing. FerrylHb is a proinflammatory agonist that targets endothelial cells. Heme release from oxidized Hb species and peroxidation of Hb are partially inhibited by Hp.

Several pathological conditions are associated with hemolysis when Hb is released from erythrocytes into the extracellular milieu [27]. Extracellular Hb exerts vasoactive effects via scavenging nitric oxide, an important vasodilator and signaling molecule (reviewed in [27]). Moreover, oxidation of extracellular Hb triggers prooxidant (reviewed in [28]) and proinflammatory effects on vascular endothelium [29]. Efficient mechanisms have evolved for the removal of Hb from the circulation to control the deleterious effects of extracellular Hb. Haptoglobin (Hp), an acute-phase protein, is present in plasma in high amounts (0.41–1.65 mg/mL) with the exclusive recognized function of capturing cell-free Hb and chaperoning Hb to macrophages for degradation (reviewed in [30]). Hp binding facilitates the removal of Hb from circulation through the CD163 macrophage scavenger receptor-mediated endocytosis [31] (Figure 1).

The formation of the Hp:Hb complex is virtually irreversible, and Hp binding besides facilitating the removal of intravascular cell-free Hb has many additional beneficial effects. Several studies showed that Hb bound to Hp is less prone to H_2_O_2_-mediated oxidation than free Hb [32–34]. In fact, the Hb:Hp complex acts as a fairly efficient peroxidase [35]. Further studies proved that Hp prevents H_2_O_2_-induced oxidation of amino acids in critical regions of Hb chains—that is, *α*Tyr42, *β*Tyr145, and *β*Cys93—and polymerization of Hb [36]. The recent determination of the crystal structure of the porcine Hp:Hb complex revealed that Hb residues known to be prone to oxidative modifications are buried in the Hp:Hb interface, thereby explaining this direct protective role of Hp against H_2_O_2_-induced oxidation [37].

Following internalization by the macrophage, heme is cleaved by heme oxygenase-1 (HO-1) into biliverdin, carbon monoxide, and iron. This mechanism not only provides effective elimination of Hb, but it also assures iron recycling for *de novo* erythropoiesis under normal circumstances. However, in cases of massive intravascular hemolysis, Hp can be depleted from the circulation in which the case of free Hb is cleared (rather inefficiently) via a low-affinity pathway through CD163 [38] and/or by renal excretion [39, 40]. This latter is accompanied by generation of free iron and organ damage.

The prooxidant effects of Hb are mainly attributed to heme release from oxidized Hb. For example, MetHb but not Hb readily releases its heme prosthetic group which can be taken up by endothelial cells [41]. As a consequence of endothelial heme uptake, cells become extremely sensitive to oxidants like H_2_O_2_ or activated inflammatory cells [41]. Endothelial cells exposed to MetHb upregulate HO-1 and ferritin, key molecules responsible for heme degradation and safe storage of iron [41]. Heme release and subsequent endothelial reactions are efficiently blocked by Hp [41]. Heme transfer from oxidized Hb toward low-density lipoprotein (LDL) and consequent lipid peroxidation is also prevented by Hp [42] (Figure 1).

However, the protective effects of Hp are not entirely straightforward. In humans, the Hp gene is polymorphic resulting in the two functional alleles, that is, 1 and 2, which can form three different genotypes: Hp1-1, Hp2-1, and Hp2-2. Because Hp1 is monovalent and Hp2 is bivalent, the structures of protein products in the three genotypes show molecular heterogeneity. Hp1-1 is a small dimeric molecule (86 kDa) whereas Hp2-1 is characterized by linear polymers (86–300 kDa) and Hp2-2 forms large cyclic polymers (170–900 kDa) (reviewed in [43]).

The Hp polymorphism was investigated as a possible genetic determinant in cardiovascular disease. These epidemiologic studies revealed that the Hp2-2 genotype is a risk factor for cardiovascular complications in both type I and type II diabetic patients (reviewed in [44]). Recently, it has been shown that Hp2-2 genotype is associated with elevated amounts of iron in atherosclerotic carotid plaques in diabetic patients [45] which may imply poor stabilization of free Hb. The Hp2-2 genotype was found to be accompanied by increased macrophage infiltration and decreased smooth muscle cell content of the atherosclerotic plaque, two common indicators of plaque instability, in patients with diabetes [46]. In healthy men, Hp2-2 genotype is found to be associated with increased circulating oxLDL levels when compared to Hp1-1 or Hp2-1 genotypes [47]. Recently, the Hp2-2 genotype has been linked to greater risk of vasospasm and of clinical deterioration by delayed cerebral ischemia following subarachnoid hemorrhage [48]. It has been shown that in preeclampsia, pathologies associated with elevated level of cell-free Hb, Hp2-1, and Hp2-2 were associated with increased plasma heme levels and decreased plasma nitrite concentration [49].

Concomitant with these clinical observations, mechanical studies were performed in order to explain the molecular basis of these genetically determined differences. It has been demonstrated that Hp1-1 more efficiently inhibits heme transfer from MetHb to LDL compared to Hp2-2 [50, 51]. It has been reported that Hp2-2:Hb complex exhibits higher functional affinity for the macrophage scavenger receptor CD163 than the Hp1-1:Hb complex [31]. In contrast, Asleh et al. found that clearance rate of Hp1-1:Hb through CD163 is much higher than that of Hp2-2:Hb [52]. There are, as well, conflicting data concerning the different antioxidant properties and different Hb scavenging capacities of Hp1-1 and Hp2-2. In a recent publication, Lipiski et al. [53] did not find differences between Hp1-1 and Hp2-2 in Hb binding and intravascular compartmentalization *in vivo*. Furthermore, heme transfer from Hb to endothelial cells and LDL were equally blocked by Hp1-1 and Hp2-2 [53]. The apparent association of the Hp2-2 genotype with more severe symptoms in different pathologies might be explained by differences in size and penetration efficiency of Hp1-1 and Hp2-2. Because Hp2-2 is a large molecule, its diffusion into deeper compartments might be limited. Clearly, further investigations are warranted to improve our understanding of the molecular basis of the epidemiological and clinical findings related to the Hp polymorphism.

Oxidation of Hb leads to the formation of structurally altered (e.g., covalently cross-linked) Hb species. It was hypothesized that these structural changes might be associated with the impairment of the endogenous scavenging pathways. Recent studies have revealed that the elimination of oxidized Hb species via both high-affinity and low-affinity pathways can be severely compromised [38, 54].

Heme can be released from Hb upon oxidation. Hemopexin (Hx) is an acute-phase plasma protein that binds heme with the highest affinity of any known protein [55]. Hx inhibits the catalytic activity of heme in oxidative reactions [56–58]. Hx-heme complexes are taken up via the scavenger receptor LDL receptor-related protein 1/CD91 [59]. Although CD91 is expressed by many different cell types, among them hepatocytes and macrophages in liver and spleen are the most important cells in scavenging the circulating Hx-heme complexes [60]. Following endocytosis, heme is degraded by HO-1 and iron is stored by ferritin [61].

Hemoglobin and heme uptake pathways as well as heme degradation and iron storage mechanisms must function efficiently to control cell-free Hb- and heme-triggered cellular and organ damage and to limit renal iron exposure/loss during hemolysis or hemorrhage. Hp knock-out and Hx knock-out mice suffer from oxidative renal injury and elevated iron loading in the kidney after a strong hemolytic stress [62, 63]. Surprisingly, Hp/Hx double-null mice show reduced Hb accumulation in the kidney and improved survival after a lethal hemolytic stimulus [64]. This suggests that other, presently uncharacterized, protective mechanisms may be present in Hp/Hx double-null mice which provide tolerance in hemolytic stress in a similar way as sickle hemoglobin confers tolerance to plasmodium infection [65].

## 5. Biological Effects of Oxidized Hb

### 5.1. Oxidized Hb as a Prooxidant

Iron compounds can facilitate the production of hydroxyl radicals from reactive oxygen species via the Fenton reaction. Sadrzadeh et al. hypothesized that Hb might provide catalytically active iron for the Fenton reaction and thus mediate hydroxyl radical generation [66]. They showed that Hb readily promotes hydroxyl radical formation in the presence of a superoxide anion-generating system [66]. They also examined the prooxidant effect of Hb on the central nervous system *in vivo*. They found that Hb injected into the spinal cord of cats mediates peroxidation of lipids in the central nervous system. The iron chelator desferrioxamine prevented the Hb-induced damage, suggesting that free iron derived from Hb/heme is the proximate toxic species [67].

Similarly, we later showed that MetHb but not Hb induces oxidative modification of LDL [42]. This effect of MetHb was inhibited by the heme-scavenging protein Hx and by Hp or cyanide, agents that either bind free heme or strengthen the heme-globin bond, highlighting the role of heme release in this process [42, 68]. Recently, we showed that interactions between Hb and lipids derived from atheromatous lesions lead to a feed-forward process of Hb oxidation: conversion of oxyHb to MetHb, spontaneous heme release, oxidative heme lysis, iron release, and further lipid peroxidation [23]. We tested whether highly oxidized forms of Hb such as ferrylHb might induce lipid peroxidation and found that ferrylHb like MetHb readily initiates oxidative modification of LDL and lipids from atheromatous lesions (Potor et al. unpublished observation) (Figure 1).

Transition metals, particularly iron, markedly potentiate oxidant damage to cells. Balla and coworkers showed that MetHb but not Hb synergizes damage from reactive oxygen species or activated inflammatory cell-mediated damage on vascular endothelial cells [41] (Figure 1). As might be expected, this effect of MetHb could be abrogated by the heme scavenger hemopexin or by strengthening the interaction between the heme and globin moieties, once again emphasizing the critical impact of heme release [41].

### 5.2. Oxidized Hb as an Inflammatory Mediator

Hemolytic or hemorrhagic episodes are often associated with inflammation even when infectious agents are absent [69]. Considerable effort has been made to define the mediators that trigger such inflammatory response. Endothelium, the interface between blood and tissue, has a pivotal role in the inflammatory response mainly by inducing the leukocyte adhesion cascade to facilitate the transmigration of inflammatory cells to the inflamed tissue. It has been shown that endothelial cells exposed to heme (100 *μ*mol/L) upregulate the expression of adhesion molecules: intracellular adhesion molecule-1 (ICAM-1), vascular cell adhesion molecule-1 (VCAM-1), and E-selectin [70]. While searching for other mediators of hemolysis-associated inflammation, we found that ferrylHb but not native Hb or MetHb triggers the upregulation of the same proinflammatory adhesion molecules. [29] (Figure 1). In fact, ferrylHb seems to be a strong agonist of this inflammatory response, because it induces adhesion molecules at concentrations as low as 10 *μ*mol/L, a concentration at which heme has no effect. Endothelial cells exposed to ferrylHb show rearrangement of the actin cytoskeleton leading to disruption of the endothelial cell monolayer, intercellular gap formation, and increased permeability of the monolayer [29]. Actin polymerization is required for ferrylHb-induced inflammatory response and involves the activation of the c-Jun N-terminal kinase and the p38 mitogen-activated protein kinase signal transduction pathways [29]. Induction of inflammation is a unique property of the heavily oxidized ferrylHb because neither Hb nor MetHb triggers these effects [29] (Figure 1).

Heme itself has been shown to induce inflammation in mice [71]. In that work, mice were injected intravenously with heme to obtain 750–1000 *μ*mol/L intravascular heme concentration (*≈*55–75 *μ*mol/kg). Heme enhanced vascular permeability and increased leukocytes migration into inflammatory areas which was accompanied by the upregulation of the adhesion molecules in liver and pancreas [71]. Heme has been shown to be chemotactic for neutrophils when is injected into the peritoneal cavity of mice at a dose of only 2–20 *μ*mol/kg [72]. We found that ferrylHb injected intraperitoneally to mice at a dose of 2 *μ*mol/kg induced a robust inflammatory response leading to the recruitment of PMN cells in the peritoneum [29]. This effect was not observed when mice were challenged with Hb or MetHb [29].

Infectious diseases that cause hemolysis are among the most threatening human diseases. Recently, new mechanisms have been proposed which might help explain why the combination of hemolysis and infection is so dangerous. It has been shown that heme, released from oxidized Hb upon hemolysis, amplifies the innate immune response to microbial molecules such as LPS [73]. This effect of heme is dependent on the generation of reactive oxygen species [73]. Moreover, heme activates toll-like receptor 4, a receptor of the innate immune system that recognizes pathogen-associated molecular patterns such as LPS [74]. In the last couple of years, heme has been implicated in different disease models (e.g., malaria and sepsis) as a molecule that can negatively modify the tolerance of the host to invading pathogens [65, 75–79].

Overall, oxidized Hb appears to be a two-edged sword, with both edges unfortunately pointed in the wrong direction. Not only does free Hb promote oxidative damage triggered by exogenous and endogenous oxidants, but highly oxidized forms such as ferryl Hb also cause the upregulation of proinflammatory adhesion molecules. The net effect of such upregulation is the recruitment of inflammatory cells such as neutrophils and macrophages with resultant promotion of local inflammatory reactions. Teleologically, such responses to oxidized Hb may make sense; the presence of a hemorrhagic focus implies that the integument has been breached. Such an incident may permit the entry of adventitious bacteria, most of which can use Hb iron as fertilizer and with which they can grow rapidly [80]. In this light, it may be no surprise that Hp can suppress the Hb-driven growth of pathogenic bacteria [44].

## 6. Conclusions

Our growing understanding of the pathophysiologic effects of extracellular Hb is leading to an improved understanding of normal and pathologic responses to various forms of Hb after release from red cells. For most of human history, the bruise was viewed as simply an interesting lesion, the color of which evolved over a period of days from red (red cells and some free oxyHb) to brown (lysed red cells, MetHb) to green/yellow (biliverdin/bilirubin). Now, it seems there may be more going on than meets the eye, especially the formation of different forms of oxidized Hb with varying biological activities. The complex system which has evolved to control and dispose of cell-free Hb generally works well unless overwhelmed by excessive hemorrhage or hemolysis. It is now becoming clear that cell-free, variously oxidized Hb is capable of promoting oxidation and inflammatory responses. This occurs through (1) the release of free heme (a toxic prooxidant molecule), (2) increasing expression of vascular adhesion molecules, and (3) the subsequent promotion of inflammatory processes. Fuller understanding of this chain of events may lead to the development of improved diagnostics and therapeutics meant to interrupt the pathologic effects of cell-free Hb.
